# An Approach for Calculating Student-Centered Value in Education – A Link between Quality, Efficiency, and the Learning Experience in the Health Professions

**DOI:** 10.1371/journal.pone.0162941

**Published:** 2016-09-15

**Authors:** Peter Nicklen, George Rivers, Caryn Ooi, Dragan Ilic, Scott Reeves, Kieran Walsh, Stephen Maloney

**Affiliations:** 1 Department of Physiotherapy, Monash University, Frankston, Victoria, Australia; 2 Department of Economics, Monash University, Caulfield, Victoria, Australia; 3 Department of Epidemiology and Preventive Medicine, Monash University, Melbourne, Victoria, Australia; 4 St. George’s University of London, Cranmer Terrace, London, United Kingdom; 5 British Medical Journal, Tavistock Square, London, United Kingdom; University Hospital Oldenburg, GERMANY

## Abstract

Health professional education is experiencing a cultural shift towards student-centered education. Although we are now challenging our traditional training methods, our methods for evaluating the impact of the training on the learner remains largely unchanged. What is not typically measured is student-centered value; whether it was ‘worth’ what the learner paid. The primary aim of this study was to apply a method of calculating student-centered value, applied to the context of a change in teaching methods within a health professional program. This study took place over the first semester of the third year of the Bachelor of Physiotherapy at Monash University, Victoria, Australia, in 2014. The entire third year cohort (n = 78) was invited to participate. Survey based design was used to collect the appropriate data. A blended learning model was implemented; subsequently students were only required to attend campus three days per week, with the remaining two days comprising online learning. This was compared to the previous year’s format, a campus-based face-to-face approach where students attended campus five days per week, with the primary outcome—Value to student. Value to student incorporates, user costs associated with transportation and equipment, the amount of time saved, the price paid and perceived gross benefit. Of the 78 students invited to participate, 76 completed the post-unit survey (non-participation rate 2.6%). Based on Value to student the blended learning approach provided a $1,314.93 net benefit to students. Another significant finding was that the perceived gross benefit for the blended learning approach was $4014.84 compared to the campus-based face-to-face approach of $3651.72, indicating that students would pay more for the blended learning approach. This paper successfully applied a novel method of calculating student-centered value. This is the first step in validating the value to student outcome. Measuring economic value to the student may be used as a way of evaluating effective change in a modern health professional curriculum. This could extend to calculate total value, which would incorporate the economic implications for the educational providers. Further research is required for validation of this outcome.

## Introduction

There is a need for improved efficiency and effectiveness of health workforce education and training to address significant workforce shortages [1]. However, the issue of efficiency needs to be considered in conjunction with the quality of the education, to ensure we are providing optimal health service delivery. Achieving an enjoyable and rewarding learning experience is also paramount for the marketability of a program, and for the pride of its educators.

Health professional education is experiencing a cultural shift towards student-centered education, which focuses on how the student understands the material [2]. This provides the learner an opportunity for greater autonomy and responsibility for their learning, and requires that the academic understands how the student learns, is invested in the process of their learning rather than the transfer of information, and is concerned about the actual process of learning happening in the students [2,3]. Greater implementation of flipped teaching, peer-assisted learning, inter-professional education, increased use of virtual learning environments and the use of simulation [4] drive the search for better teaching, better learning. Although we are now challenging our traditional training methods, through adopting new technologies and pedagogies, our methods for evaluating the impact of the training on the learner remains largely unchanged. Evaluation of teaching units is important for informing the ongoing search for quality, ensuring competitiveness amongst educational providers, particularly within undergraduate physiotherapy programs in Australia, and ensuring program sustainability and accountability in an increasingly challenging economic climate. Student feedback has been a key focus in the evaluation of programs, units, and its teachers [5]. What is not typically measured is student-centered value; whether it was ‘worth’ what the learner paid, and using this measure to inform change in practice.

Value can be interpreted as a ratio, or trade-off, between quality and price [6,7,8,9]. Value has been widely accepted in retail for some time with Hartnett [10] highlighting, for example, that retailers who meet customer needs, are delivering value, which places them in a stronger position long term. Value to students represents the price paid, additional expenses categorized into user costs, and the perceived gross benefit. However, it is important to note that ‘good value’ can apply to both high and low cost items, as long as the consumer feels that their perceived gross benefit, including their learning outcomes and learning experience, are matched by the cost of participation, the relationship of quality and price [6]. Tailoring the use of educational technologies and other innovative teaching methods for maximum institutional benefit may risk a perception of cost-cutting from a student perspective, typically associated with a decrease in the quality of the education [11,12]. This is a poor motivator for academics focused on educational excellence. In contrast, tailoring the use of educational technologies for the maximum value to the learner becomes a marketable educational strength.

The concept of value of education is particularly pertinent in the field of health professional education [13], due to its relatively high cost of delivery by the provider, and of participation by the learner. TTThis expense stems from the educational methods utilized in health professional education, including the small group learning and workplace integrated learning environments traditionally utilised to meet the levels of accountability and scrutiny required to ensure the competence of our health workforce. This is accentuated by the tendency for health professional education to maintain traditional teaching practices, rather than challenging our teaching approaches, due to a master-apprentice culture in which we have learned that we should teach through the same methods that we were taught. Even though there is some literature that examines the concept of value from the perspective of the provider [14,15] as of yet, little published literature exists investigating the concept of educational value in economic terms from the perspective of the student [12]. The lack of literature is perhaps ironic given that healthcare has been one of the most fertile grounds for cost-effectiveness and other economic evaluations of its interventions and processes [13,16].

The purpose of this novel study was to apply a method of calculating student-centered value, in the context of challenging the space and mode of learning.

## Aims

1. To apply a method of calculating student-centered value, applied to the context of a change in teaching methods within a health professional program.
2. Explore the impact of modifying the mode and space of undergraduate education in a health professional training program on learning outcomes and quality.

## Methods

### Design

The study involved the determination of student value. A one-off, cross-sectional study design was implemented for initial data collection, with economic modeling subsequently applied. Ethics approval for the study was obtained through the Monash University Human Research Ethics Committee (MUHREC) (Ethics CF14/307–2014000115).

### Justification of Context

In order to evaluate the central aim of this study, being the application of an economic method for calculating 'value to student', the study required an appropriate context to apply the economic modeling. The essential ingredients of an appropriate context to evaluate the method included 1) A sizeable change occurred in the teaching pedagogy, 2) that the students experienced the pedagogy before and after the change, 3) that further 'traditional measures' of success of the teaching pedagogy were available in order to assist in validating the approach, and 4) that the pedagogy change analysed was authentic and relevant to contemporary issues in health professional education.

The context chosen that met all of these criteria was the third year, first semester of a campus-based 24-credit point unit, within a physiotherapy program based at Monash University, Victoria, Australia, in 2014. During this period there was a significant shift in the unit’s scheduled teaching and learning activities, from a campus based face-to-face to a blended learning format. The research team was able to proactively engage with the teaching staff to ensure that only the method of delivery and no other changes (i.e. learning outcomes or assessments) were made during the study period. The physiotherapy course applied standardised teaching processes and activities across the first three years of the program, enabling the reference point for the students’ experience of the incumbent pedagogy. The unit has consistent baseline measurements of student grades, and pre-existing validated measures of student experience. In Australia, students are able to pay university fees upfront, or if eligible, receive a government loan to cover the cost of university fees, which is then paid back.

This unit is the final campus-based unit prior to clinical placements. It is made up of a ten-week teaching calendar. Previous to 2014, students completed this ten-week on-campus five days per week. This was made up of a range of learning activities including campus-based lectures, face-to-face case based learning (CBL), practical sessions and face-to-face tutorials.

### Unit Changes—Implementing a Blended Learning Model

In 2014, many of the learning activities that made up the previous unit were moved to a web-based environment. The learning objectives of the unit at the past and present time-points remained unchanged, and all assessment tasks and formats were retained. Changes were only made to the mode (e.g. web-based versus face-to-face), and space (e.g. student were not restricted to campus). The 2014 unit still had a ten-week teaching calendar, however, students were only required to attend campus three days per week, with the remaining two days comprising online learning as a replacement to the previous campus-based learning activities. The major structural changes included the implementation of web-conferencing CBL, 50% reduction of lectures and introduction of online lectures, increased use of low-fidelity and high-fidelity simulations, structured self-directed practical sessions supplemented by eBook creations, reducing live tutor attendance in practical activities by 50%, the implementation of peer-assisted tutoring, and an increase in online self-directed learning resources. Improving the learning experience, overcoming challenges of space, and providing greater flexibility were all factors that drove the changes to the unit.

### Participants

The entire 2014 third year cohort (n = 78) was invited to participate. In order to meet unit requirements, all students had to complete the learning activities. An independent research assistant recruited participants through face-to-face delivery and distribution of an information package, which included the explanatory statement. The explanatory statement made it clear that students who choose not to consent to the study were not required to complete the surveys relating to this study. All data collected was non-identifiable and students were assured that those that did not consent could not be identified. This method of consent was approved by MUHREC.

### Outcomes

#### Primary Outcome—Value to Student

**Fig 1:**
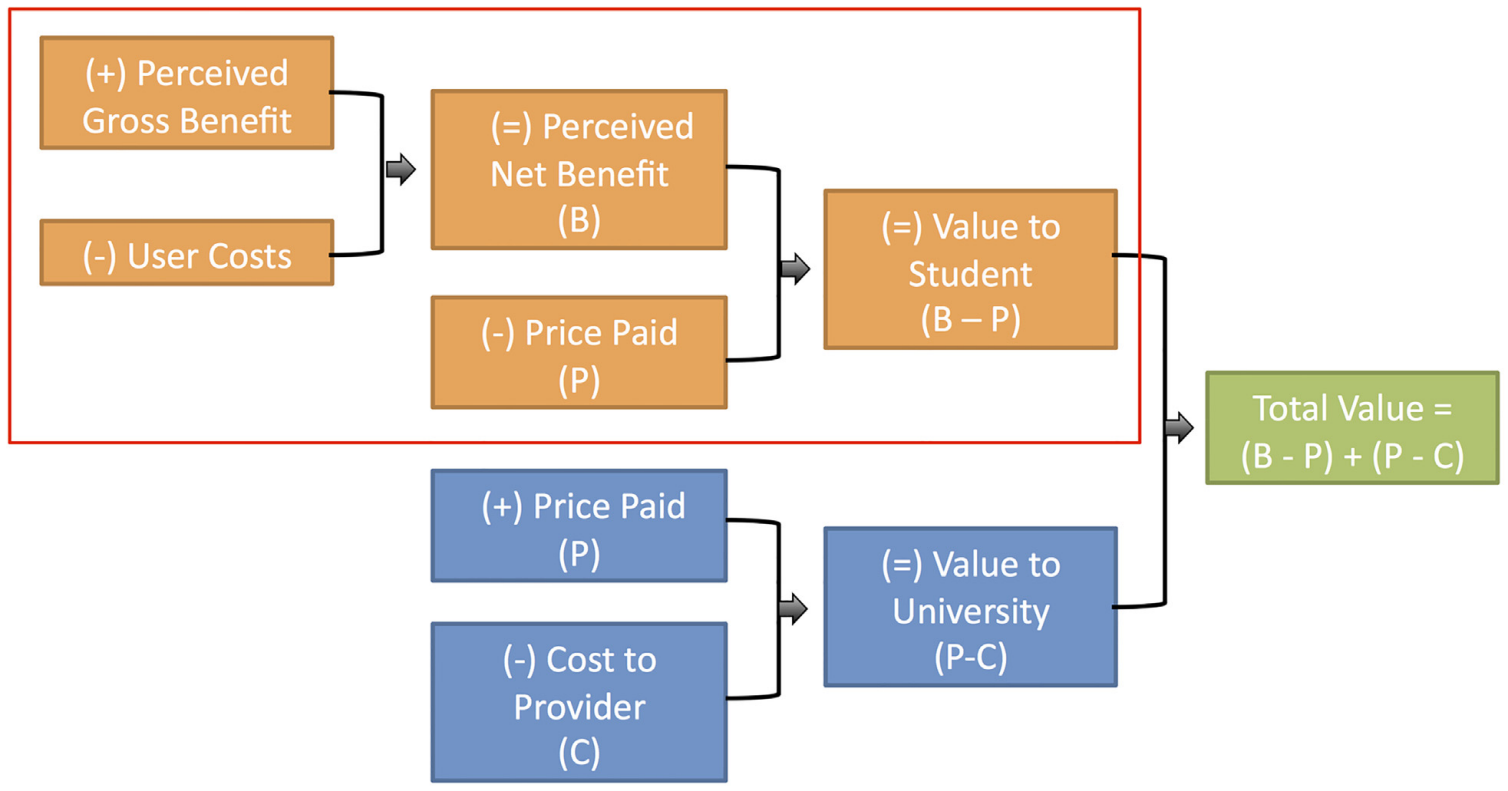
Determine Total Value. The red box highlights the focus of this study.

Total value is regarded as the sum total of value to consumer (student) and value to provider (university). The focus of this study is the value to student (Fig 1). This represents the price paid, in this context the course fees, and perceived net benefit, which is made up of user costs and perceived gross benefit. These data were collected via a post-unit survey. Data collected from the 2014 third year cohort was used to estimate value to student of the third year first semester unit for both 2013 and 2014, given these students have completed the previous four 24-credit point units comprising of standardized campus-based face-to-face learning methods, establishing their prior teaching and learning experiences on which to base the comparison. All outcomes were distributed and collected by an independent research assistant.

#### Secondary Outcomes

Secondary outcomes were used to validate value to student. This included the traditional methods of unit evaluation and performance, including written unit examination results and components of the student evaluation of teaching and units (SETU). Mean and standard deviation was used given the continuous nature of the data. Scores were compared between the 2013 third year cohort (the previous unit—campus-based face-to-face learning) and 2014 third year cohort (the current unit—blended learning). The written unit examination included multiple choice, short answer and open-ended questions. These were sampled from the unit learning objectives. The median value of 10 items were extracted from both the 2013 and 2014 SETU responses, which covered student satisfaction and quality of both learning and teaching. These were scored on a 5-point Likert scale, which ranged from strongly disagree to strongly agree, with 5 representing the maximum/positive pole. The median was used given the categorical nature of the data.

### Data Analysis

Two methods of data analysis were utilised to determine the value to student. Each method compared this value between the previous and current unit structure to determine which had greater value to the student. The first method involved averaging each data point to determine the mean value to student. The second method involved determining each student’s individual value and identifying the median, this was used to validate the result of the first method.

#### Key data points

Perceived Gross Benefit represents the price the student would pay for that model of teaching. Participants were asked how much they would pay for the current unit structure (blended) and how much they would pay if they completed the third year first semester unit as per the previous unit structure (campus-based face-to-face). This was represented via box plot analysis with percentiles. Participants were also asked to estimate user costs under the following domains; time costs for both attendance and travel, travel related costs including tyre and service costs, fuel, tolls and parking permits, cost relating to web-based learning including hardware costs, electricity and internet use, and food expenditure. Time cost for both attendance and travel was estimated based on participants’ reported current average hourly rate. This was then multiplied with both reported travel time and designated class time. Tyre and service, and fuel costs were estimated based on reported costs from the RACV Motoring Cost Reports from 2011 and distance traveled as reported by students. Perceived Net Benefit was determined by subtracting the user costs from the perceived gross benefit. Price Paid was estimated by averaging the reported course fees on three comparable university websites each providing undergraduate physiotherapy courses, one from Victoria, one from NSW and one from Queensland. Value to Student was then determined by subtracting the price paid from the perceived net benefit Eq (1).

Value to Student=(Perceived Gross Benefit−User Costs)− Price Paid (1)

The two unit structures were then compared based on value to student and the difference was evaluated. A sensitivity analysis was completed to determine what factors influence the value to student the greatest. This involved manipulating individual data points to determine their effect on the overall result. Switching values were calculated for the key factors—perceived gross benefit and user costs, to determine what value was required to change the direction of the result between the previous unit structure and blended learning structure.

#### Determining User Costs (Mean)

User costs were determined firstly by estimating costs of a single day per student, for each of the three possible situations, 1) Previous Unit Structure (Face-to-face learning), 2) Blended Learning Format—On campus learning, 3) Blended Learning Format—Off campus learning. Within each possible situation a separate user cost was determined for each different mode of transport (car, public transport, walk). A weighted sum was determined for each situation accounting for the number of students who travels by each mode of transport Eq (2).

Weighted sum=(user costs [drive] × (n [drive]÷n[total]))+ (user costs [public transport] × (n [public transport]÷n[total]))+ (user costs [walk] × (n [walk]÷n[total])) (2)

where n = number of students

This weighted sum represents the average student’s daily costs. The weighted sum was then used to determine the total cost for week and then semester Eq (3).

User costs for semester=[weighted sum]×[number of days]×[number of weeks] (3)

### Secondary Outcomes

Unit exam results and SETU scores were presented with means and standard deviations. Standardized effect size (Cohen’s d) and 95% confidence interval (C.I) was calculated to compare results from 2013 and 2014. Results were considered statically significant if the confidence interval did not include zero.

## Results

### Participant Information

All 78 students enrolled in the third year of a Bachelor of Physiotherapy in 2014, were invited to participate. All students completed the required learning activities in order to successfully complete the unit. There were no dropouts during the semester. Of the 78 students, 76 completed the post-unit survey (non-participation rate 2.6%).

### Primary Outcome Value to Student—Mean

**Fig 2:**
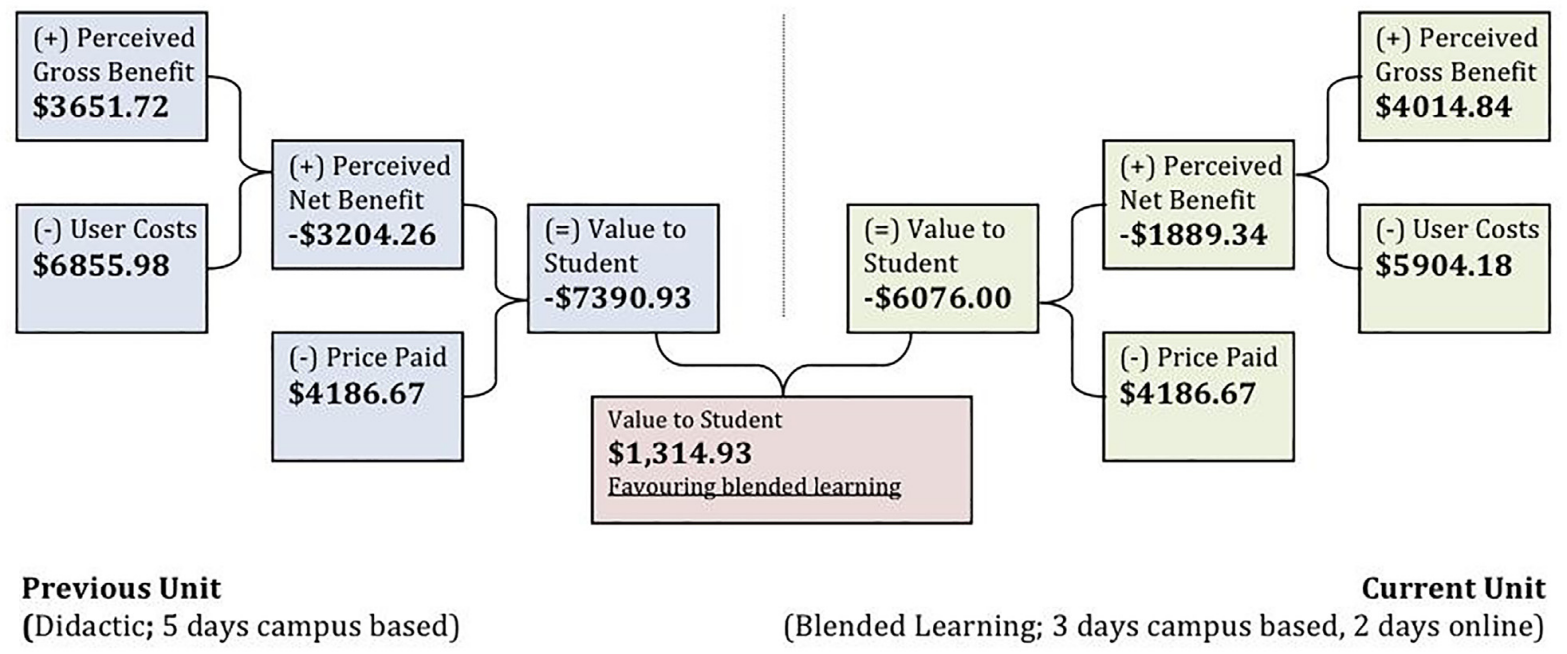
Summary of Mean Value to Student.

Based on value to student, the blended learning changes (current unit) within the unit provided students with a net incremental value of $1,314.93 when compared to the campus-based face-to-face approach (previous unit) (Fig 2).

#### Perceived Gross Benefit

**Fig 3:**
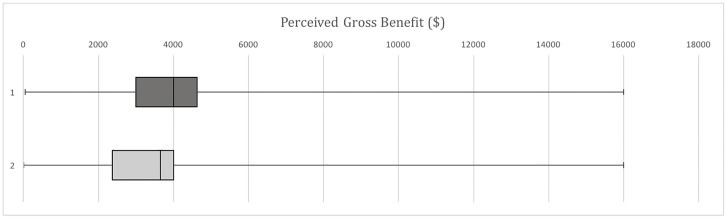
Comparison of Perceived Gross Benefit. Key: 1 = Current Unit Format; 2 = Previous Unit Format. 5-number summary (min, 1st quartile, median, 3rd quartile, max): Current Unit Format = 50, 3000, 4000, 4625, 16000; Previous Unit Format = 10, 2375, 3650, 4000, 16000.

Perceived Gross Benefit was compared between the current unit format and previous unit format (Fig 3).

#### User Costs

The weighted sum was used to determine the total user cost for semester per student (Table 1). Time costs were greater for the current unit compared to the previous unit on campus, due to the increased number of face-to-face hours on averages hours per day. Public transport costs were considered, however, all participants that stated they caught public transport reported using the free university shuttle service.

$$\begin{array}{l} \text{User cost for previous unit }=\text{ weighted sum }\times \;\left[\text{no}.\text{ of days}\right]\;\times \;\left[\text{no}.\text{ of weeks}\right]\;\\ =\;137.13\;\times \;5\;\times \;10\;=\;\$6855.98 \end{array}$$

$$\begin{array}{l} \text{User cost for current unit }=\text{ (}\left[\text{weighted sum }\times \text{ no}.\text{ of days}\right]\;+\text{ [weighted sum }\\ \times \text{ no}.\text{ of days}])\times \;\left[\text{no}.\text{ of weeks}\right]\;=\;\left(142.79\;\times \;3\;+\;81\;\times \;2\right)\;\times \;10\;=\;\$5904.18 \end{array}$$

**Table 1:** Estimating User Costs.

	Previous unit (on campus)	Current unit (on campus)	Current unit (off campus)
**Total user costs ($) (per semester per student)**	6855.98	5904.18	
Mode of transport	Drivers = 63; public transport = 4; walkers = 9		
Number of hours per week	17	11	6
Average hourly rate	21.80	21.80	21.80
**Time costs per day ($)** (attendance and travel)	99.09	104.79	65.41
**Travel costs per day ($)** (tyre, servicing, fuel, tolls)	37.29	37.29	0.00
**Parking permit per day ($)**	0.68	0.68	0.00
**Hardware and utilities per day ($)**	0.86	0.86	6.37
**Food costs per day ($)**	5.68	5.69	9.24
**Weighted** Sum ($) (per day)	**137.13**	**142.79** (on campus)	**81.00** (off campus)

### Value to Student—Median (Individual Data)

Individual data points were estimated for each student as per the above method to determine individual value to student. The median of this data was then found.

$$\text{Median value to student }=\underline{\text{\$1215}.\text{34 per semester in favour of blended learning }\left(\text{current unit}\right)}\;$$

### Missing Data Calculation

12 students did not report perceived gross benefit and therefore individual value to student was unable to be determined. This meant that these students’ data was removed from the second method of data analysis—median value to student.

### Switching Value

In order to reach equivalent value to student and switch the direction of the result, perceived gross benefit for the previous format would need to increase to $4,966.64, with the current format needing to decrease to $2,699.92. The user costs for the previous format would need to decrease to $5,541.05 and the user costs for the current format would need to increase to $7,219.11.

### Summary of Unit Results

The unit exam result was comparable between the previous unit and the current, blended learning unit (Mean (SD) Previous Unit (%) 75.97 (9.56) n = 73; Current Unit (%) 76.74 (6.57) n = 78, effect size (C.I) = 0.09 (-0.23–0.41) (Table 2).

**Table 2:** Summary of Secondary Outcomes.

	Previous unit—campus-based face-to-face	Current unit—blended learning	Effect size (C.I)*
Unit exam result mean (SD) (%)	75.97 (9.56)	76.74 (6.57)	0.09 (-0.23–0.41)
SETU results av. median (SD) (scored out of 5)	4.25 (0.12)	4.62 (0.22)	1.98 (1.51–2.42)

Key: SETU response options 1 = strongly disagree, 2 = disagree, 3 = neutral, 4 = agree, 5 = strongly agree.

*Effect sizes < .3 are considered small, .3>.8 are considered moderate, and >.8 are considered large

#### Student Evaluation of Teaching and Units Results

The median of 10 items were taken from the Student Evaluation of Teaching and Units (SETU) to compare the previous unit to the current unit. Response rate for the previous unit was 61.6% (n = 45/73) and for the current unit 88.5% (n = 69/78). All 10 items increased in favour of the blended learning unit and the mean was statistically significant (mean (SD) Previous Unit = 4.25 (0.12); Current Unit = 4.62 (0.22); effect size (C.I) = 2.09 (0.93–3.07) (Table 2).

## Discussion

This research aimed to apply a method of calculating student-centered value, in the context of challenging the space and mode of learning. The findings indicate that students valued the blended learning approach more. This is supported by secondary outcomes, suggesting that learning outcomes are equivalent with both approaches, based on the unit examination. Student satisfaction was also greater with the blended learning approach compared to the campus-based face-to-face approach.

Within the context of this study, two teaching approaches based on the concepts of value, price and worth were compared. Value to students incorporates the price paid, user costs, and the perceived gross benefit. The perceived gross benefit encompasses both perceived value and student satisfaction. Zeithaml [17] and later Sweeny [6] suggested that perceived value is the consumer’s assessment of the usefulness of a product or service, or its ability to meet needs and wants, based on perceptions of what is received and what is given. Quality and price are two important components of perceived value for money [6]. Given students had experienced both approaches of teaching prior to data collection, the perceived gross benefit also encompasses an element of student satisfaction. This means that the magnitude of value to students represents actual savings with the comparison of user costs, student satisfaction and perceived worth with the comparison of perceived gross value and difference in the price of the service.

Value is an important concept within education. Given the shift towards student-centered learning within higher education, success should be measured on student learning and depth of understanding, rather than how many of the learning objectives were covered by the academic [2]. Academics must consider learning experiences and learning outcomes, alongside measures of cost and value when reviewing practices; value to student and furthermore total value allows this union to occur. The cost and value of teaching and learning practices in medical education directly impacts on the accessibility, efficiency and quality of education [18,19,20]. Education of optimal value to the student, may have flow on benefits to the university and the health sector through improved student recruitment, through the promotion of student diversity, and improved learning outcomes. The concept of student-centered economics of learning may be used to evaluate teaching units and assist in the decision-making processes. Furthermore, it may motivate academics to challenge their traditional practices and subsequently drive curriculum (re)development and change to teaching and learning practices. This could decrease risk at an administrative level and potentially impact the accessibility of education and improve return on investment for students.

The effect on learning outcomes should be a consideration with any change in education. Therefore, it was important to us to ensure the structural changes to the unit and implementation of blended learning did not have a negative effect on student learning. Given all 10 items on the SETU increased from 2013 to 2014, it could be assumed that satisfaction was higher in the blended learning unit compared to the campus-based face-to-face approach. These results further support the notion that value to students incorporates an element of student satisfaction, as education that provides increased satisfaction is of greater value to students, which is reflected in the perceived gross benefit. This result may be attributed to the mode of learning, students simply preferring to learn via web-based learning, however this could also be due to the flexibility of web-based learning or due to the reduction in campus time. It is also possible that students’ preference the blended learning model due to the novelty of the approach, however, in other pilot studies by the research team, students have preferred face-to-face learning over blended learning models. We believe this may be due to the implementation and student orientation process having been refined and improved, subsequently increasing student satisfaction with the blended learning model.

Given the paucity of literature on value from the student perspective, this paper does not consider value to the provider. It is important to recognise that value from the student perspective and from the university perspective could be considered alongside each other to strengthen current economic-analysis methods within education. In order to incorporate value to the provider alongside measures of student value, the price paid by consumers and cost to the provider needs to be considered (Fig 1). Cost to the provider might include such things as staffing costs in regards to planning, marking, teaching and administration, space costs, and other resources such as equipment.

Both of the approaches to calculate value to students have strengths and limitations. The first method, which determined the mean value to student using averages, was much more user-friendly requiring less time to complete the calculation. The second approach, which determined the median value to student, arguably has more scientific rigor from an economic research perspective, however it is less likely to be adopted and applied due to the additional steps and time required. One limitation of this faster ‘field test’ method is that of the aforementioned missing data, which either needs to be estimated or the participant results need to be excluded, which could potentially decrease the validity of the results.

The study contains a number of limitations that may influence the strength of the findings. Data for the economic evaluation was collected from the 2014 cohort; this was used to estimate user costs and perceived gross benefit for both approaches. The secondary outcomes where compared between the 2013 and 2014 cohorts, which means this data does not directly correlate with the economic data, however it is likely to be an appropriate estimation. We feel this is probably a fair comparison given the high level of consistency in teaching methods and unit format in previous units experienced by the 2014 cohort. It is also possible that there is an unintended difference in the level of difficulty in the 2013–2014 examinations, although this is considered during the examination writing stages. This model was applied to a unit of physiotherapy, therefore, further research is required to determine whether it can be applied other programs.

## Conclusions

This paper successfully applied a novel method of calculating student-centered value, in the context of challenging the space and mode of learning. This is the first step in validating the value to student outcome. Measuring economic value to the student could be used as a way of evaluating effective change in a modern health professional curriculum. The approach provides a more real-life context, including the costs of participation and the students’ perception of benefit received from the education. This could extend to calculate total value, which would incorporate the economic implications for the educational providers. The findings within the context of this study indicate that the blended learning approach provided a greater value to the students, which was supported by the secondary outcomes of student learning and satisfaction. Further research is required for validation of this outcome.

## Supporting Information

S1 File Raw data sets. (XLS)
